# Unlimited access to health care - impact of psychosomatic co-morbidity on utilisation in German general practices

**DOI:** 10.1186/1471-2296-12-51

**Published:** 2011-06-18

**Authors:** Antonius Schneider, Elisabeth Hörlein, Eva Wartner, Isabelle Schumann, Peter Henningsen, Klaus Linde

**Affiliations:** 1 Institute of General Practice, Klinikum rechts der Isar, Technische Universität München, Orleansstrasse 47, 81667 München, Germany; 2 Institute of Psychosomatic Medicine, Klinikum rechts der Isar, Technische Universität München, Ismaninger Strasse 22, 81675 München, Germany

## Abstract

**Background:**

The effect of psychosomatic co-morbidity on resource use for systems with unlimited access remains unclear. The aim of this study was to evaluate the impact on practice visits, referrals and periods of disability in German general practices and to identify predictors of health care utilisation.

**Methods:**

Cross sectional observational study in 13 practices in Upper Bavaria. Patients were included consecutively and filled in the Patients Health Questionnaire (PHQ). Numbers of practice visits, referrals and periods of disability within the last twelve months and permanent mental and somatic diagnoses were extracted manually by review of the computerised charts. Physicians in Germany are obliged to document repetitive reasons of encounter as permanent diagnoses in terms of ICD-10-codes. These ICD-10-codes are used for legitimisation of reimbursement in German general practices.

**Results:**

1005 patients were included (58.6% female). On average, patients had 15.3 (sd 16.3) practice contacts, 3.8 (sd 4.2) referrals and 7.5 (sd 23.1) days of disability per year. The mean number of coded permanent diagnoses was 0.4 (sd 0.7) for mental and 4.0 (sd 4.0) for somatic diagnoses. Patients with mental diagnoses scored higher in depression, anxiety, panic and somatoform disorder scales of PHQ. Frequent practice visits were associated stronger with coded permanent mental diagnoses (OR 20.0; 95%CI 7.5-53.9) than with coded permanent somatic diagnoses (OR 14.4; 95%CI 5.9-35.4). Frequent referrals were associated stronger with somatic diagnoses (OR 4.9; 95%CI 2.0-11.9) than with mental diagnoses (OR 3.6; 95%CI 1.4-9.8). Periods of disability were predicted by mental diagnoses (OR 5.0; 95%CI 1.6-15.8) but not by somatic diagnoses (OR 2.5; 95%CI 0.7-8.1).

**Conclusions:**

Psychosomatic co-morbidity has a stronger impact on health care utilisation in German general practices with respect to practice visits and periods of disability whereas somatic disorders play a stronger role for referrals. Time constraints in the practices might lead to frequent contacts as too little time is left for patients with mental problems. Therefore, structural changes in the health care reimbursement systems might be necessary. Mental diagnoses might be helpful to identify patients at risk for high health care utilisation. However, the use of routinely coded diagnoses for reimbursement might lead to distorted estimation of resource use.

## Background

Depression, anxiety and somatisation are the most common mental disorders in primary care with prevalence estimates between 10% and 20% [1,2]. It has been shown that psychosomatic disorders lead to higher utilisation of health care resources [3-5] which is in particular true for somatisation disorders [6-8]. However, most studies evaluating the impact of psychosomatic co-morbidity on health care utilisation are performed in health care systems with limited access to specialised disciplines [5,6]. E.g., high utilisers were defined as patients who visit the practice 4.55 times per year as an average [6] which is due to the gate keeping system. The effect of psychosomatic co-morbidity on health care systems with unlimited access remains unclear. Ambulatory health care in Germany is mainly provided by private for-profit providers, including general practitioners and almost all specialists. Physicians receive reimbursement per each quarter. Beyond that, there is no formal gate keeping role for general practitioners in Germany which allows unrestricted numbers of referrals to specialists per year. The sole restriction is that only one referral to a certain specialist (e.g. one cardiologist, one orthopaedic etc.) within three months is allowed. In addition patients had to pay one-time €10 for the first physician contact in each quarter, with many exceptions for that (like having chronic illness, participation in disease management programs). It has been reported that a German general practitioner (GP) has about 242 patient contacts per week which is twice the number as in other countries like UK, USA or the Netherlands [9].

The impact of psychosomatic co-morbidity on the medical care utilisation has been investigated in the German National Health Interview and Examination survey [3,4]. However, information on the number of practice visits, periods of disability and days in hospital within the last twelve months was collected from patient questionnaires, which might lead to distorted estimation of medical care utilisation. We aimed to investigate the impact of psychosomatic co-morbidity on practice visits, referrals and periods of disability which were objectively derived by chart review.

## Methods

### Study design

This cross sectional observational study was performed in a sample of patients, which were all insured by a statutory health insurance, from thirteen general practices in the Region of Upper Bavaria in Southern Germany. The study was approved by the Medical Ethics Committee of the Technische Universität München/Klinikum rechts der Isar. The study was carried out between April and August 2010. All participants provided written informed consent.

All consecutive adult patients who attended their physician in the study period were asked in the waiting room by the research assistants (EH, EW) to fill in a questionnaire before seeing the doctor. Patients below the age of 18 years and patients with poor German language skills were excluded. The number of practice contacts, number of referrals and periods of disability within the last twelve months were derived by chart review from the computerised medical record systems. Additionally, all permanent diagnoses for each patient were extracted manually and documented in a structured form. The GPs are obliged to document repetitive reasons of encounter as permanent diagnoses in terms of ICD-10-diagnoses in their medical records as they are used as a legitimisation for reimbursement.

### Measures

The scales of the PHQ questionnaire were used to estimate the adequacy of the coded diagnoses of the physicians. The patients filled in the questionnaire before attending their physician. The depression severity score comprises nine items which can be summarized. The range is from 0 (no depression) to 27 (maximal depression). Superior criterion validity of the PHQ compared to other established self-report questionnaires was confirmed with respect to the diagnoses of 'major depressive disorder' and 'other depressive disorders' made by a standard interview in assessing psychiatric disorders [10,11]. The PHQ anxiety disorder section measures panic disorder and other anxiety disorder. The panic disorder score comprises five items. According to DSM-IV diagnostic algorithms, panic disorder is diagnosed when all 5 PHQ items are answered positively whereas the other anxiety disorder section primarily includes 7 criteria for generalized anxiety disorder. Excellent operating characteristics have been demonstrated for the American [12,13] and German versions of the PHQ [10,11]. For simplicity, a score was calculated by addition of the single scores. The range was from 0 (min.) to 14 (max.) for anxiety disorder and 0 (min.) to 15 (max.) for panic disorder. Somatisation was measured using the somatic symptom module of the PHQ [14]. The PHQ-15 has high internal reliability and construct validity [14]. The PHQ-15 inquires about 15 somatic symptoms or symptom clusters that account for more than 90% of the physical complaints reported in the outpatient setting and includes 14 of the 15 most prevalent DSM-IV somatisation disorder somatic symptoms [14]. A somatisation severity score can be derived by inclusion of two questions of the depression module. The range is from 0 (no somatisation) to 30 (severe somatisation). The score was shown to be sensitive for the course of the disease, and was demonstrated to be useful for estimation of health care utilisation [15].

### Analysis

Patients' characteristics were analysed descriptively. Diseases which are coded as permanent diagnoses varied widely. Categories of mental disorders (e.g. depression, anxiety disorder, somatisation disorder, fatigue syndrome, addiction), somatic disorders (e.g. hypertension, coronary artery disease, diabetes, asthma) and the combination of both categories were established. To investigate differences between females/males and participants/non-participants the t-test or the χ^2^-test was used as appropriate. For comparisons between diagnostic categories the χ^2^-test, the Kruskal-Wallis test or analysis of variance was used. High utilizers were defined as patients whose number of practice visits, referrals or periods of disability exceeded the median values of patients without permanent diagnosis of mental disorders. A multivariate binary logistic regression model was calculated to determine the strongest predictors of health care utilization. Number of practice visits, referrals and periods of disability were established as dependent variables. The permanent somatic and mental diagnoses, age, sex and education were included as independent variables into this model which was corrected for the interaction of permanent somatic and mental diagnoses. As the distribution of the number of permanent somatic diagnoses was skewed, somatic diagnoses were binary coded (lower vs. higher than median). Mental diagnoses were coded binary as 'no mental diagnosis' vs. 'at least one mental diagnosis'.

## Results

A total of 1341 patients were invited to participate in the study and 1011 (75.4%) agreed. The permanent diagnoses of six patients were not documented in the charts. 58.6% of the remaining 1005 patients were female and 65.8% had an educational level lower than final secondary-school examinations (Table 1). Participants were significantly younger than non-participants (mean 49.3 vs. 59.1 years; p < 0.001), there were no sex differences. The mean number of practice visits per year was 15.3 (sd 16.3), the mean of referrals was 3.8 (sd 4.2) and periods of disability for the whole sample were 7.5 (sd 23.1) days. 349 (34.7%) patients received a certificate of incapacity for work. The mean of periods of disability for this group was 20.0 (sd 34.3) days (not in table). All distributions were strongly skewed with most patients having low values and some patients having very high values. For reasons of simplicity we present means and standard deviation throughout this manuscript.

**Table 1:** Characteristics of patients with numbers of practice visits, referrals and work leaf within 12 months in relation to documented continuous diagnoses (n = 6 are missing; p-values are related to Chi-square test (sex, education), analysis of variance (age) or Kruskal-Wallis test (utilisation measures).

Permanent diagnoses		Female	Age	Education ≤ 10 years	Practice visits within 12 months	Referrals within 12 months	Periods of disability within 12 months
	
	N (%)	n (%)	mean (sd)	n (%)	mean (sd)	mean (sd)	mean (sd)
None	159 (15.8)	83 (52.2)	33.5 (12.3)	78 (49.1)	4.4 (4.1)	1.1 (2.0)	4.4 (7.8)
≥ 1 mental	35 (3.5)	18 (51.4)	38.6 (9.8)	26 (74.3)	23.9 (27.4)	2.5 (2.4)	8.1 (11.4)
≥ 1 somatic	562 (55.9)	321 (57.1)	52.9 (17.5)	372 (66.2)	13.6 (10.6)	3.9 (4.0)	6.4 (23.1)
≥ 1 mental and ≥ 1 somatic	249 (24.8)	166 (66.7)	52.7 (15.7)	183 (73.5)	24.6 (22.8)	5.3 (5.0)	11.6 (29.0)
All patients	1005 (100)	588 (58.5)	49.3 (17.7)	659 (65.6)	15.2 (16.3)	3.8 (4.2)	7.5 (23.12)
p-value		0.004	p < 0.001	p < 0.001	p < 0.001	p < 0.001	p < 0.001

**Figure 1:**
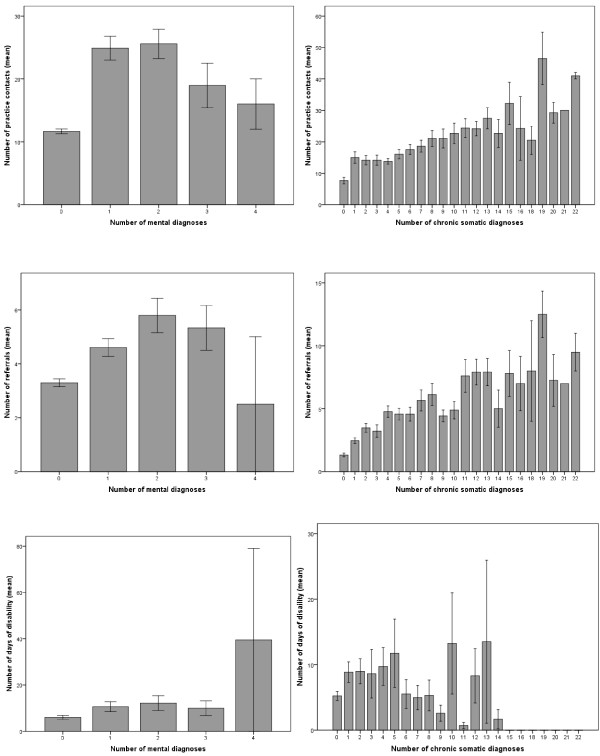
**Relation between permanent diagnoses and number of practice visits, referrals and days of disability within twelve months**. Values are mean (SEM)

284 (28.3%) patients received at least one permanent mental diagnosis. The mean of coded mental permanent diagnoses was 0.4 (sd 0.7), with a maximum of four diagnoses. 811 (80.7%) patients received at least one permanent somatic diagnosis. The mean of coded somatic permanent diagnoses was 4.0 (sd 4.0) with a maximum of twenty-two diagnoses. Patients with a permanent diagnosis of mental disorder only or both mental and somatic diagnoses had significantly more practice visits, more referrals to specialists within the last 12 months and a higher number of days of disability compared to patients without documented permanent diagnoses (Table 1). The number of practice visits, referrals and periods of disability increased with the number of coded somatic diagnoses; the association with mental disorders was not linear (Figure 1). Patients with permanent diagnoses of mental disorders scored significantly higher in all scales relating to depression, anxiety, panic and somatoform disorder followed by those patients suffering from both chronic mental and physical diseases (Table 2).

**Table 2:** Depression, anxiety, panic and somatoform scales of the Patients Health Questionnaire in relation to documented continuous diagnoses (p-values are related to Kruskal-Wallis-test)

Permanent diagnoses	Depression	Anxiety	Panic disorder	Somatoform disorder
	mean (sd)	mean (sd)	mean (sd)	mean (sd)
None	4.60 (3.98)	3.73 (2.77)	0.59 (2.26)	4.80 (3.50)
≥ 1 mental	10.69 (7.18)	7.20 (3.90)	4.93 (5.47)	8.05 (4.81)
≥ 1 somatic	4.76 (4.13)	3.94 (2.90)	0.81 (2.63)	5.19 (3.78)
≥ 1 mental and ≥ 1 somatic	7.48 (5.39)	5.82 (3.47)	2.22 (4.13)	6.96 (4.40)
All patients	5.61 (4.82)	4.48 (3.22)	1.26 (3.27)	5.67 (4.04)
p-value	p < 0.001	p < 0.001	p < 0.001	p < 0.001

The median of practice visits of patients without permanent diagnosis of mental disorders was 11 times per year; the median of referrals was 3, and the median of periods of disability was 10 days. The median of coded permanent somatic disorders was 3. The binary logistic regression showed that high utilisation with respect to practice visits was associated strongest with permanent mental diagnoses, shortly followed by somatic diagnoses (Table 3). High utilisation of referrals was associated stronger with somatic diagnoses than with mental diagnoses. Periods of disability were predicted by mental diagnoses whereas somatic diagnoses were not significantly associated. The interaction analysis between the permanent mental and somatic diagnoses was significant with respect to number of practice visits. Therefore, a detailed analysis was performed for patients with and without permanent diagnoses of somatic disorders. In this analysis permanent mental diagnoses were strongly associated with practice visits in patients without permanent somatic diagnoses (OR 16.0; 95%CI 6.3-40.7; p < 0.001), whereas the predictive value was lower when the patients had at least one permanent somatic diagnosis (OR 2.5; 95% CI 1.8-3.5; p < 0.001) (data not in table). Beyond that, the number of practice visits and referrals increased and periods of disability decreased with age. Female gender was associated with more referrals and lower periods of disability. Lower educated patients had more practice visits and more periods of disability.

**Table 3:** Odds Ratios (ORs) for medical care utilisation from multivariate binary regression analyses

	Practice visits > 11 per year		Referrals > 3 per year		Periods of disability > 10 dy	
	OR (95%CI)	p	OR (95%CI)	p	OR (95%CI)	p
At least one permanent mental diagnosis	20.0 (7.5-53.9)	< 0.001	3.6 (1.4-9.8)	0.011	5.0 (1.6-15.8)	0.007
> 3 permanent somatic diagnoses	14.4 (5.9-35.4)	< 0.001	4.9 (2.0-11.9)	< 0.001	2.5 (0.7-8.1)	0.143
Female	1.2 (0.9-1.7)	0.185	1.4 (1.1-1.9)	0.016	0.7 (0.5-1.0)	0.043
Age	1.0 (1.0-1.0)	< 0.001	1.0 (1.0-1.0)	< 0.001	1.0 (1.0-1.0)	< 0.001
Education ≤ 10 years	1.6 (1.2-2.2)	0.006	0.8 (0.6-1.1)	0.206	2.3 (1.5-3.7)	< 0.001

> 11 practice visits, > 3 referrals, > 10 days of disability is defined as high utilisation (cut-off by the median)

Multivariate ORs are adjusted for the interaction term of mental and somatic diagnoses

## Discussion

Our study results show a generally high health care utilisation with more than 15 practice contacts and three to four referrals on the average per year. Consistent with previous reports, we found that high medical service utilization is associated with prevalent mental disorder. Beyond that, our findings indicate that the influence of psychosomatic disorders on utilisation appears to be stronger than the influence of purely somatic disorders.

The impact of psychosomatic or mental disorders on health care utilisation is already well described for gate keeping systems [5,6]. We found such an effect also in our survey. However, in our study high utilisation was defined when patients were coming more than eleven times per year into practice, which is more than twice as much compared with an US study [6]. Beyond that, we confirmed a very high rate of practice contacts per year in general which is twice as high as in the US or the Netherlands [9]. These facts might be due to free access of health care and the rewarding system. In Germany a physician receives reimbursement per quarter only if the patient comes within the respective quarter. A previous German survey [3,4] found only 3.8 practice visits of patients with and 2.7 visits of patients without mental disorders; and they found only 12.9 days of disability. However, the information about health care utilisation in that survey was assessed by self-rating which might have led to distorted estimation, whereas our information was gathered by review of the computerised charts. Detailed analyses of our study demonstrated that especially permanent mental disorders were leading to high practice contact rates, referrals and periods of disability. The impact of psychosomatic diagnoses was stronger than that of permanent somatic diagnoses in general with exception for referrals. These results illustrate that the coding of permanent mental disorders might allow identifying patients at risk for high utilisation during medical encounter. Identification of difficult patients is valuable not only regarding resource use but in particular as high utilisation is associated with harmful side effects [16,17]. However, our findings highlight that the management of patients with psychosomatic or mental problems is a big challenge in German general practices. On the one hand, these patients are frequent attenders. On the other hand, it seems likely that there is not enough time for them due to the high contact rate. Therefore, structural changes in the health care reimbursement systems might be necessary to give physicians more time to actively detect and manage patients with mental problems. This might be useful as several patient-centred communication strategies have been shown to improve the management of patients with high psychosomatic co-morbidity, like the intervention of an extended reattribution and management for patients with functional disorders in general practice [8,18].

The documentation of permanent diagnoses has a crucial role in the German health care system as it is the precondition for payment of specific diagnostic and therapeutic procedures. This role will become even more relevant in future as insurances have to invest more in sicker patients. It is assumed by health care managers that the morbidity is adequately reflected by ICD-10 diagnoses which are coded in general practices. Therefore, practitioners will have to document each reason of encounter as an ICD-10 diagnosis for each quarter. However, the concept of ICD-10-documentation is not without drawbacks. In primary care it is often impossible to establish a diagnosis for each reason of encounter. Beside that, practitioners might be tempted to document questionable diagnoses for ensuring their salary. Furthermore, our findings suggest that the mental diagnoses have a more profound impact on practice visits and periods of disability than the somatic diagnoses. This was especially true for the periods of disability which were not even significantly associated with the number of somatic diagnoses. This might underline that exaggerated coding of diagnoses does not necessarily lead to a comprehensive understanding of the patients' condition and the associated resource use on practice level. In fact, such coding might lead to distorted estimation of resource use.

The reliability of the permanent diagnoses has to be discussed as a limitation. It is unlikely that the mental diagnoses have been made exactly according to the ICD-10 classification. The related diagnostic algorithms are complex and might be not always suitable for primary care [19,20]. Patients with coded mental diagnoses scored significantly higher in all PHQ scales relating to depression, anxiety, panic and somatoform disorder indeed. It might be a limitation that coded diagnoses may contain past diagnoses that the patient is not suffering from now. However the clear differences of the PHQ scales suggest that the coded diagnoses might have been updated in some degree. For example, patients with mental disorders showed PHQ values representing at least moderate depression [15]. Adding the PHQ anxiety and panic items to a summary score is not commonly used. The GAD-7 was shown to be suitable for continuous measurement [21], but this questionnaire was not available to us when the study was established. However, the scales are similar; and the sum of the PHQ items would be lower than the GAD-7 items. This would even have led to an underestimation of the anxiety and panic scores. The validity of the permanent somatic ICD-10-diagnoses might be questioned as other coding systems like ICPC-2 (International Classification of Primary Care) are supposed to be more valid for research purposes in primary care [22,23]. However, there is also conflicting evidence regarding the diagnostic precision of ICPC-2 [24]. Nonresponder were significantly older than responder. This might lead to an underestimation of the frequency of practice contact, referrals and periods of disability. However, it is unlikely that this affects our findings on the role of psychosomatic comorbidity.

## Conclusions

Psychosomatic co-morbidity has a strong impact on health care utilisation in general practices and might explain in parts the high number of practice visits, referrals and periods of disability in Germany. Time constraints in the practices might lead to frequent contacts as too little time is left for patients with mental problems. Therefore, structural changes in the health care reimbursement systems might be necessary. Permanent mental diagnoses might be helpful to identify patients at risk for high health care utilisation. However, the use of routinely coded diagnoses for reimbursement might lead to distorted estimation of resource use.

## Competing interests

The authors declare that they have no competing interests.

## Authors' contributions

AS designed the study, and wrote the manuscript. EH and EW recruited the patients and helped with data management and interpretation. IS helped to perform the survey and with writing. PH helped with interpretation of the data and writing the manuscript. KL helped with statistics and writing. All authors read and approved the final manuscript.

## Pre-publication history

The pre-publication history for this paper can be accessed here:

http://www.biomedcentral.com/1471-2296/12/51/prepub
